# Effects of Efficient Ethylene Remover on the Lignification of Fresh Faba Bean (*Vicia faba* L.) during Storage

**DOI:** 10.3390/foods13193036

**Published:** 2024-09-25

**Authors:** Jiaxing Fan, Cunkun Chen, Xiaojun Zhang, Chenghu Dong, Manqin Jin, Xuemei Zhang, Wentong Xue, Jingming Li

**Affiliations:** 1 College of Food Science and Nutritional Engineering, China Agricultural University, Beijing 100083, China; fanjiaxing0902@163.com (J.F.); 13161183611@163.com (X.Z.); jinmanqin0618@163.com (M.J.); zxmgr@cau.edu.cn (X.Z.); xwt@cau.edu.cn (W.X.); 2 Institute of Agricultural Products Preservation and Processing Technology (National Engineering Technology Research Center for Preservation of Agriculture Product), Tianjin Academy of Agricultural Sciences, Key Laboratory of Postharvest Physiology and Storage of Agricultural Products, Ministry of Agriculture of the People’s Republic of China, Tianjin Key Laboratory of Postharvest Physiology and Storage of Agricultural Products, Tianjin 300384, China; cck0318@126.com (C.C.); dongchenghu2022@126.com (C.D.)

**Keywords:** postharvest, storage quality, key enzymes, lignification process, transcriptomics

## Abstract

Postharvest ethylene accumulation and lignification are significant issues affecting the storage quality of fresh faba beans, resulting in rapid quality decline. However, there is still a lack of effective preservation methods to preserve the quality of faba beans during storage. This study aimed to investigate the regulation of lignification in faba beans during storage using a high-efficiency ethylene remover (HEER), examining physiological responses, key enzyme activities, and transcriptomic changes. Results showed that the HEER treatment inhibited the lignification, reducing it by 45% and lowering the respiratory rate of fresh pods by 32.8% during storage. Additionally, the HEER treatment suppressed respiration rates and the activities of lignin synthesis-related enzymes, including phenylalanine ammonia-lyase (from 353.73 to 246.60 U/g), cinnamic acid-4-hydroxylase (from 635.86 to 125.00 U/g), 4-coumarate: coenzyme A ligase (from 1008.57 to 516.52 U/g), and cinnamyl-alcohol dehydrogenase (from 129.42 to 37.12 U/g), thus slowing lignin accumulation. During storage, the hardness of fresh faba bean increased by 9.79% from the initial period, being 1.44 times higher than that of HEER. On days 8 and 16 of storage, the respiratory rate of the treated beans decreased by 24.38% and 4.12%, respectively. Physiological and enzyme activity analyses indicated that HEER treatment-induced increase in hardness was associated with the phenylpropanoid metabolic pathway. Moreover, the HEER significantly down-regulated the expression of several key genes, namely *FaPAL*, *FaC_4_H*, and *FaCAD*. This study helps to deepen the understanding of the inhibition of lignification by HEER and provide new insights for the development of preservation technology of faba bean.

## 1. Introduction

Faba bean (*Vicia faba* L.) is the third largest legume and is widely cultivated in North and South America, the Mediterranean region, Australia, China, West Asia, and Europe [1] Fresh faba beans are considered to be the most promising legume due to the increasing demand for healthy food and the need for sustainable development of the food system [2]. They are richer in protein, fiber, and carbohydrates compared to other beans. Additionally, fresh faba bean seeds contain high levels of vitamin C, iron, zinc, and selenium [3,4]. However, harvested faba beans have a short shelf life as they are prone to yellowing and aging. In particular, the lignification of fresh faba beans leads to the hardening and roughness of the tissue during storage, ultimately reducing their commercial value.

Lignin is the second most abundant polyphenolic polymer in higher plants, typically associated with tissue hardness. The phenylpropane metabolic pathway plays a crucial role in lignification as it is primarily responsible for producing secondary metabolites, including lignin, in plants. This pathway begins with the deamination of phenylalanine to form cinnamic acid, which then undergoes a series of hydroxylation, methylation, and reduction reactions to produce five major monomers for lignin biosynthesis. The main processes involved in lignification are the biosynthesis, transport, and polymerization of lignin monomers, each resulting from the coordinated expression of numerous enzyme genes within the phenylpropanoid pathway. Several enzymes have been identified as key players in postharvest lignification. For example, phenylalanine ammonia-lyase (PAL), cinnamic acid-4-hydroxylase (4CL), cinnamate-4-hydroxylase (C_4_H), and cinnamyl-alcohol dehydrogenase (CAD) crucial in this process [5]. Research on Pyrus *pyrifolia Whangkeumbae* species has revealed that enzymes such as PAL, 4CL, and CAD exhibit divergent expression patterns during lignification [6,7,8,9]. Additionally, the expression patterns of *PpCAD1* and *PpCAD2* are positively correlated with hard-end fruit lignin content during fruit development and cold storage periods. An increase in synthesis-related enzymes such as PAL, 4CL, CAD, and POD accelerates cellulose and lignin synthesis, promoting greater levels of bamboo shoot lignification [10,11]. Furthermore, in the retardation of the lignification process in common bean using 1-MCP, enzymes related to lignin synthesis, including PAL, 4CL, and CAD, were inhibited, which reduced the rate of lignin synthesis [12].

Ethylene plays a crucial role as a plant hormone in various aspects of plant growth and aging. When fruits and vegetables are stored in confined spaces postharvest or undergo mechanical damage during harvesting, they produce a significant amount of ethylene, which is not easily evaporated. This accelerates the maturation and aging process of the plants, leading to a reduction in storage quality [13]. Therefore, reducing the release of ethylene from fruits and vegetables or decreasing their exogenous ethylene content can effectively delay the aging process without compromising their quality characteristics [14]. In fresh produce, when the cumulative ethylene content within the packaging reaches 1ppm, fruits and vegetables will further ripen and reduce their shelf life [15]. Tzeng et al. [16] found that by incorporating a potassium permanganate-based ethylene scavenger into banana packaging, the reduction of TSS content could be mitigated and the manifestation of browning could be postponed. Additionally, it was observed that HEER had the potential to delay nutrient loss and improve the storage quality of tomatoes and mangoes [17].

Controlling the release of ethylene has been found to be beneficial in improving the postharvest storage quality of fruits and vegetables. Xie et al. found that treating fresh common beans with different concentrations of 1-MCP efficiently decreased their respiration rate, thereby prolonging the storage life, especially at a concentration of 0.5 mL/L [18]. Similarly, the application of chitosan led to the formation of a thin film on the surface of Cucumis melo, which effectively reduced the respiration rate, decreased ethylene release, inhibited fruit ripening and senescence, and maintained the postharvest quality of the fruit [19]. However, while current research focuses on extending storage periods by utilizing ethylene gas for this purpose, fewer studies address lignification through the efficient removal of ethylene gas, and virtually none pertain to faba beans.

The objective of the present study was to investigate the effect of ethylene on lignification in faba beans during cold storage. The focus was on evaluating the activity of enzymes related to lignin metabolism, including PAL, C_4_H, 4CL, and CAD, as well as the expression of the genes *FaPAL*, *FaC_4_H*, *Fa4CL*, and *FaCAD*. This study enhances our understanding of the mechanisms regulating lignin accumulation in faba beans treated with ethylene.

## 2. Materials and Methods

### 2.1. Samples Collection and Preparation

Fresh faba beans were harvested on 16 August 2020, in Lanzhou, Gansu Province, China. All samples were transported to the laboratory on the same day of harvesting using fresh air. The selected beans were uniform in size and free from diseases, with fresh pods that were full of seeds and free from black spots or mechanical damage. After pre-chilling at a temperature of 0 °C ± 1 °C for a duration of 12 h, the beans were packaged with microporous material, each basket contains a 2.5 kg sample of beans. The inner dimensions of the basket are 35.5 cm × 28 cm × 10.5 cm. The beans were packaged with microporous material O_2_ permeability: 248,000 cm^3^/m^2^·24 h·1 atm; CO_2_ permeability: 256,000 cm^3^/m^2^·24 h·1 atm; moisture permeability at 50% RH, 23 °C). The control group received no treatment (0 g HEER), while the treatment group received 5 g of HEER with a ratio of 1:5. The main ingredient in HEER, potassium permanganate (KMnO_4_) in powder form (Tianlong Inc., Xi’an, China), which is packaged in a sachet (these sachets must have high permeability to gaseous ethylene, while being impermeable to water). The purity of KMnO_4_ is 99% analytical reagent grade. It showcases scavengers containing approximately 4–6% KMnO_4_ on an inert substrate. Sampling and observation were conducted at 0, 4, 8, 16, 24, and 32 days, with the initial value recorded on day zero serving as a reference point. All experiments were repeated three times to ensure accuracy and consistency.

### 2.2. Weight Loss

The weight loss of the faba bean was assessed using Tokala’s method [20]. All data were derived from the mean of three replicates. The weight loss was calculated using the following formula Equation (1).

Weight loss (%) = (Initial weight − Final weight)/(Initial weight) × 100(1)

### 2.3. Color

The color of fruits and vegetables was measured at thirty random faba bean pods on the surface of each faba bean using a fully automatic colorimeter (CM-5 type, KONICA MINOLTA, Shanghai, China). The total color difference ΔE was calculated according to Equation (2) [21].

ΔE = √([(L* − L0)]^2^ + [[(a* − a0)]^2^ + (b* − b0)]^2^), (2)

where L*, a*, and b* represent the color indices of samples at different storage times, while L0, a0, and b0 denote the color indices of faba bean.

### 2.4. Hardness

Hardness was measured using a TA. XT plus texture analyzer (TA. XT plus, Stable Micro Systems Ltd., Surrey, Godalming, UK) with a 75 mm diameter probe. Measurements were taken at the equatorial position of each faba bean. Ten parallel measurements were conducted for each treatment group, and the results were presented as mean values. Hardness values were presented as mean ± standard deviation (SD).

### 2.5. Respiration Rate

Respiration rate was measured every 8 days up to 32 days using a gas analyzer (O_2_/CO_2_ Gas Composition Detector, Tiandi Shouhe Ltd., Beijing, China), as described by Zhang et al. [22]. All data were calculated as the mean of three replicates and presented as the mean ± SD.

### 2.6. Vitamin C (VC)

VC contents were determined following the method described by Wani et al. [23]. Faba bean tissue samples (1.0 g) were added to 2 mL of pre-cooled 1 M acetate solution and homogenized with 2 mL of 1 M acetic acid solution. The mortar was then rinsed with additional 1 M acetic acid solution, and the homogenized sample, along with the wash solution, was transferred to a 10 mL centrifuge tube. The mixture was ground to homogeneity in an ice bath, followed by centrifugation at 4 °C (10,000× *g*) for extraction for 20 min. The VC content was measured at 590 nm, and the results were expressed as mg/g.

### 2.7. Lignin, Total Phenols, Flavonoids

Lignin, total phenols, and flavonoid contents in faba bean were analyzed using a test kit (Solarbio Technology Co., Ltd., Beijing, China). Each treatment group was measured three times in parallel. The results were recorded as mean ± SD, expressed in mg/g.

### 2.8. Activities of the Phenylalanine-Ammonia-Lyase (PAL)

PAL contents were determined following the method of Cao et al. [24]. Faba bean tissue samples (5.0 g) were homogenized in an extraction solution (0.05 M, pH 8.8, boric acid-borax buffer) and then centrifuged at 4 °C (12,000× *g*) to obtain the supernatant. The resulting supernatant was mixed with 3 mL of reaction fluid (0.05 M, pH 8.8, boric acid buffer) and 0.5 mL of L-alanine solution (0.02 M), and the absorbance at a wavelength of 290 nm was measured. One unit (U) of PAL activity was defined as the increase in absorbance at 290 nm by one per minute, and the results were expressed as mean ± SD.

### 2.9. Activities of Cinnamyl Alcohol Dehydrogenase (CAD), Cinnamic Acid-4-Hydroxylase (4CL), Cinnamic Acid 4-Hydroxylase (C_4_H)

The activities of CAD, 4CL, and C_4_H in faba bean were determined using a test kit (Solarbio Technology Co., Ltd., Beijing, China). Each treatment group was measured three times in parallel.

### 2.10. RNA Extraction and Transcriptomics Detection

The total RNA from the faba bean was isolated using a Tsingke plant RNA kit (Tsingke Biotechnology Co., Ltd., Beijing, China), and cDNA was synthesized using Adama’s life mix (Adama’s life Ltd., Shanghai, China). Q-PCR was determined using the 2× Color SYBR Green q-PCR Master Adamas Life, Europe). Primer sequences are shown in Table 1. The relative expression levels of genes were calculated according to the 2^−ΔCt^ method using the β-actin gene as a target gene. Three independent biological replicates were performed in the analysis.

### 2.11. Statistical Analysis

The data were presented as mean ± standard deviation. Data presentation was performed using GraphPad Prism 8 (San Diego, CA, USA). Correlation analyses were plotted using OriginLab (Northampton, MA, USA). Significance (* *p* < 0.05, ** *p* < 0.01) was determined by Student’s *t*-test.

## 3. Results and Discussion

### 3.1. Physiological Indicators of Bean Pods Following Different Treatments

**Figure 1:**
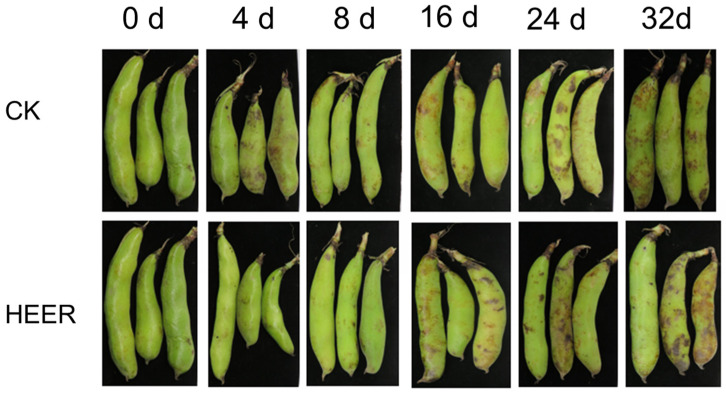
The effects of different treatments on the storage stability of faba bean pods.

The effects of different treatments on the storage ability of faba beans are shown in Figure 1. HEER effectively preserved the vibrant green hue of fresh pods, indicating its capability to retard the aging process of faba bean pods.

**Figure 2:**
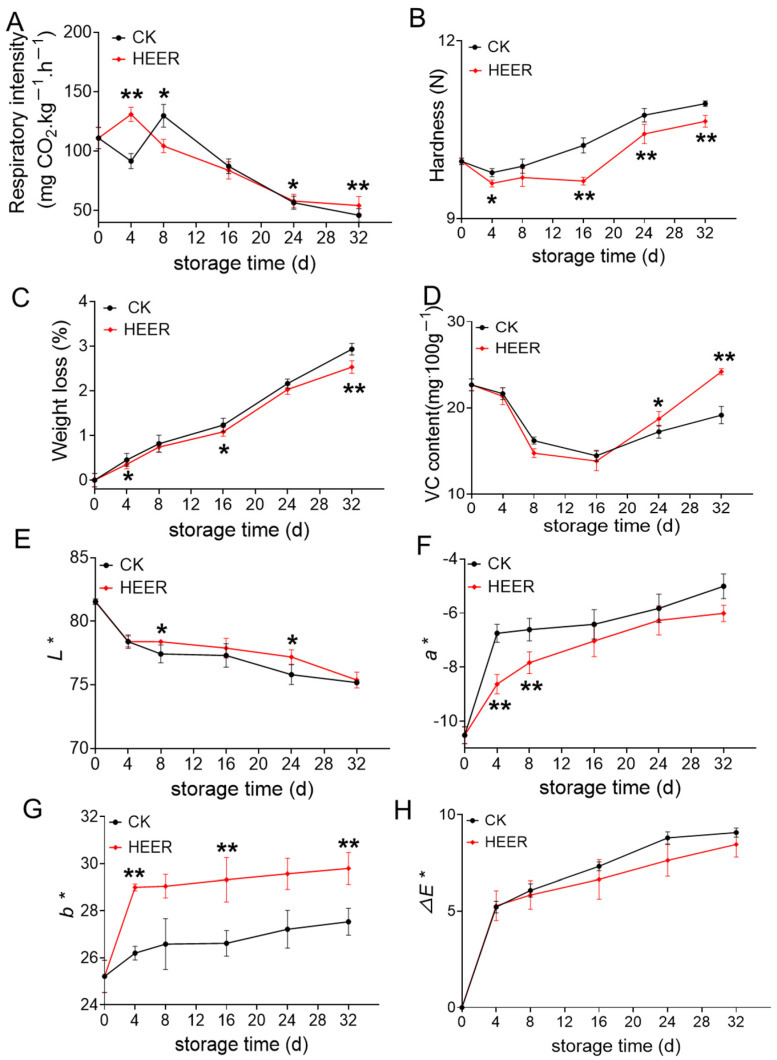
General quality characteristics of faba beans stored under different doses of HEER (0 to 32 d). (The main ingredient in HEER is potassium permanganate (KMnO4) in powder which is packaged in sachets (these sachets must have high permeability to gaseous ethylene while being impermeable to water). The purity of KMnO_4_ is 99% for analytical reagent grade. It showcases scavengers containing approximately 4–6% KMnO_4_ on an inert substrate.) Respiratory rate (**A**), hardness (**B**), weight loss (**C**), VC content (**D**), L* (**E**), a* (**F**), b* (**G**), ∆E* (**H**) (* *p* < 0.05, ** *p* < 0.01).

Respiration rate is a crucial indicator of the postharvest physiological status of vegetables and fruit [25]. The effect of HEER treatment on the respiratory rate of faba bean during storage was investigated, as shown in Figure 2A. The respiratory rate of the HEER treatment group showed fluctuations during storage, with a peak at day 4 (130.97 mg CO_2_·kg^−1^·h^−1^) in the HEER group and at day 8 (129.70 mg CO_2_·kg^−1^·h^−1^) in the CK group, potentially due to an accelerated respiratory peak induced by HEER treatment. However, over the entire storage period, the respiratory rate decreased by 24.38% at day 8 and by 4.12% at day 16 compared to that of the CK, indicating that HEER treatment significantly inhibited the faba bean respiration. This finding aligns with observations in apples and apricots treated with ethylene postharvest. Tokala et al. [20] observed a reduction in respiration rate for Cripps Pink and Granny Smith apples following ethylene treatment. Liang et al. [26] also reported similar effects for apricots.

Mechanical damage to faba beans during harvesting causes cell wall polymer splitting and vascular bundle disorganization, leading to cellulose aggregation and affecting cell wall stiffness [27,28]. As shown in Figure 2B, the hardness of fresh faba beans increases during storage, but HEER treatment delays this increase, possibly due to reduced weight loss. The CK group showed higher weight loss compared to the HEER group during storage. Additionally, PAL activity and lignin content are lower in the HEER compared to the control level. Furthermore, Liu et al. [29] and Chen et al. [30] reported that ethylene treatment slows down the reduction in fruit hardness by promoting lignin accumulation. Moreover, Cai et al. [31] observed increased flesh hardness and toughness, along with higher activities related to lignin synthesis (CAD and PAL), in postharvest loquat fruits.

VC is a crucial indicator of the nutritional value of fruits and vegetables. As shown in Figure 2C, the weight loss of faba beans increased with prolonged storage due to respiration and transpiration, by which the beans consume water and nutrients [32,33]. Figure 2D indicated that VC contents initially decreased and then increased during storage. Compared to the control level, the HEER treatment showed a faster decrease in VC content by 9.8% and 4.50% at 8 and 16 days, respectively, indicating that vitamin loss is related to the ripening process. Tissue damage and disease during maturation release oxidants, leading to VC breakdown to prevent the degradation of these oxidizing substances. Consequently, VC content increases in the later stages of storage [34].

Color is a key indicator of faba bean quality. As shown in Figure 2E, the L* value of fresh faba beans decreased during storage, reducing surface brightness, while a* and b* values increased, indicating a shift from bright green to light green. The ∆E* value demonstrated that the colorimetric changes induced by HEER treatment are not significantly different from those observed in the control group CK.

### 3.2. Inhibition of Stimulated Metabolites by HEER

**Figure 3:**
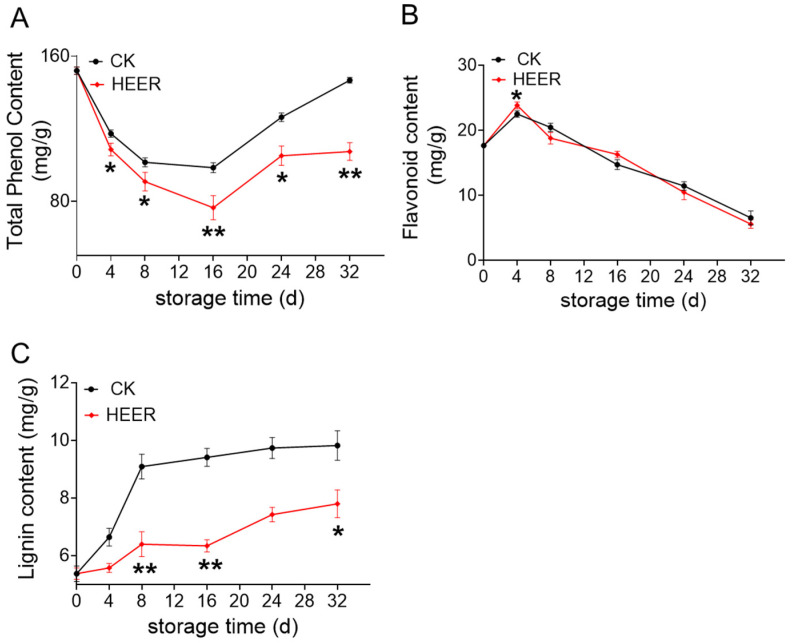
Total phenol content (**A**), flavonoids content (**B**), and lignin content (**C**) of faba beans during storage under different doses of HEER. (The main ingredient in HEER is potassium permanganate (KMnO_4_) in powder which is packaged in sachets (these sachets must have high permeability to gaseous ethylene while being impermeable to water). The purity of KMnO_4_ is 99% for analytical reagent grade. It showcases scavengers containing approximately 4–6% KMnO_4_ on an inert substrate.) (* p < 0.05, ** p < 0.01).

The total phenol content in fresh faba beans initially decreased and then increased during storage (Figure 3A). After 16 days, there was a rapid increase, potentially due to heightened PAL enzyme activity accelerating phenolic synthesis. By the end of the 32-day storage period, the total phenolic content was 146.74 mg/g for the CK group and 107.44 mg/g for the HEER group. As faba bean pods age, phenolics and pectin enhanced cell wall interactions, potentially decreasing phenolic content. Both HEER and CK groups showed increasing polyphenol content after 32 days, indicating continued aging. This suggested ethylene impeded the ripening process, consistent with similar results in ethylene-treated tomatoes [35]. Enzymatic browning or lignin monomer synthesis may have led to greater phenolic utilization compared to synthesis via alternative pathways [36].

As illustrated in Figure 3B, the flavonoid content in faba beans showed an initial increase followed by a decrease, with significant differences between the CK and HEER treatments. Figure 3C showed that the lignin content in fresh faba beans steadily increased over the storage period, affecting the texture of fruits and vegetables, which is in line with the lignification process observed in bamboo shoots, green asparagus, and courgette during storage [37,38]. Particularly, after 32 days of storage, the CK group had a lignin content of 9.82 mg/g, while HEER had 7.80 mg/g compared to their initial levels. The changes in lignin content correlated with the hardness of faba bean samples, indicating that increased lignin directly influenced firmness. The HEER treatment inhibited lignin accumulation. Similar trends have been reported in crops like glutinous maize, bamboo shoots, and kiwifruit, where hardness and lignification increase during storage, consistent with the finding in a previous study [39,40].

### 3.3. Inhibition of Four Terminal Key Enzymes by HEER

The process of lignification involves the synthesis and polymerization of lignin-associated enzymes and the expression of corresponding genes [41]. Numerous previous studies have identified PAL, C_4_H, and 4CL as key enzymes in the phenylpropane metabolic pathway [42]. PAL initiates lignification by deaminating L-phenylalanine [43], while 4CL converts 4-coumaric acid to coumaroyl coenzyme A, a fatty acid derivativeL [44]. Additionally, CAD plays a crucial role in catalyzing the final step of monolignin biosynthesis. Its activity is primarily associated with the precursors involved in lignin synthesis—the building blocks for lignin monomers [45].

**Figure 4:**
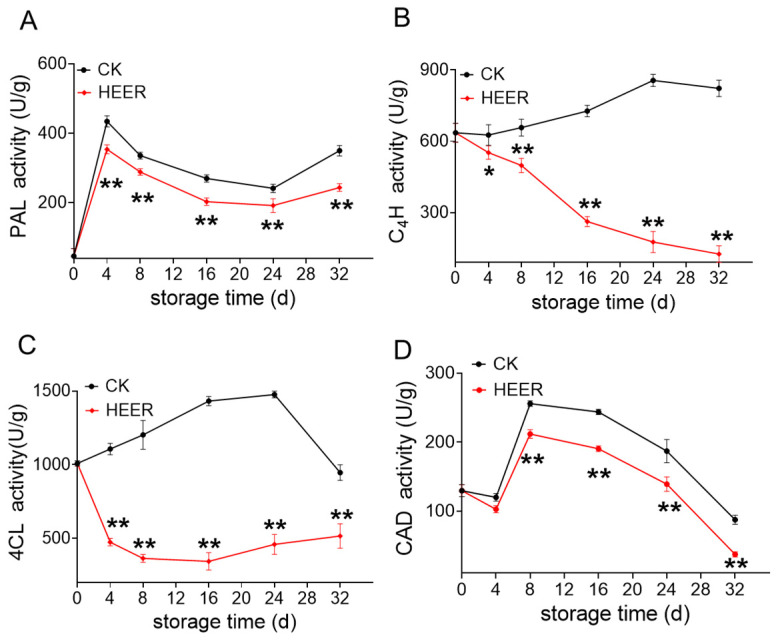
Enzyme activity in faba beans during storage under different doses of HEER. PAL (**A**), C_4_H (**B**), 4CL (**C**), and CAD (**D**). (The main ingredient in HEER is potassium permanganate (KMnO4) in powder which is packaged in sachets (these sachets must have high permeability to gaseous ethylene while being impermeable to water). The purity of KMnO_4_ is 99% for analytical reagent grade. It showcases scavengers containing approximately 4–6% KMnO_4_ on an inert substrate.) (* p < 0.05, ** p < 0.01).

This study examined the activities of lignin biosynthesis-related enzymes and gene expression during faba bean storage. PAL plays a crucial role in the phenylpropane pathway, which is essential for lignin production in plants [46,47]. PAL activity in fresh faba bean reached its peak at day 4, then decreased, with HEER treatment inhibiting its activity after 16 days (Figure 4A). This aligns with findings on delayed lignification in water bamboo shoots, where PAL, cinnamyl alcohol dehydrogenase (CAD), peroxidase (POD), and laccase activities were inhibited [48]. The C_4_H activity increased during later storage stages but decreased at day 4 in the HEER group (Figure 4B). In comparison to the control group, 4CL activity was suppressed initially during storage but gradually increased after 16 days (Figure 4C). The rate of decline in CK accelerated between day 24 and day 32 but remained higher than that in the HEER-treated samples. CAD, crucial for lignin monomer synthesis [49], increased during the first 4 days, then gradually decreased. HEER-treated samples showed suppressed CAD activity throughout storage (Figure 4D). The postharvest application of HEER inhibited the accumulation of lignin content in fresh faba bean by suppressing the activities of PAL, C_4_H, 4CL, and CAD, which is consistent with studies on fresh kidney bean, asparagus, and kiwifruit [50].

**Figure 5:**
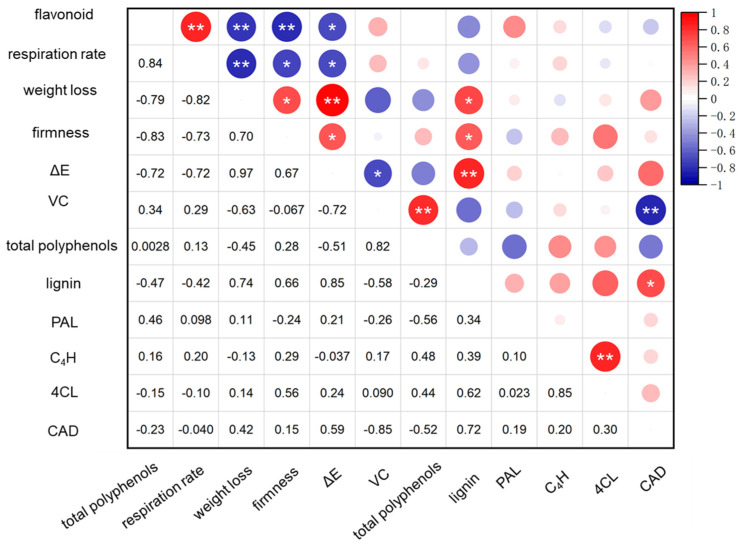
Linear correlation analysis was conducted to investigate the relationship between enzyme activities and physiological indices in postharvest faba bean treated with HEER. (The main ingredient in HEER is potassium permanganate (KMnO4) in powder which is packaged in sachets (these sachets must have high permeability to gaseous ethylene while being impermeable to water). The purity of KMnO_4_ is 99% for analytical reagent grade. It showcases scavengers containing approximately 4–6% KMnO_4_ on an inert substrate.) (* *p* < 0.05, ** *p* < 0.01).

Correlation analysis revealed a positive correlation between weight loss, lignin content, and color in HEER-treated faba beans, aligning with the finding by Jiang et al. [51] who demonstrated a similar effect in maintaining vibrant color in nectarines by reducing weight loss. Changes in respiration rate and VC content were positively correlated with flavonoids and total phenols. Additionally, 4CL and CAD enzyme activities were positively associated with C_4_H and lignin content. Previous studies on bamboo shoot lignification also linked lignin accumulation to increased PAL and CAD activities [48]. Conversely, changes in respiratory rate, weight loss, hardness, and VC content negatively correlated with elevated weight loss, flavonoids, and CAD (Figure 5). These results suggested that HEER inhibited the respiratory rate, delaying weight loss, and inhibited 4CL and CAD activities, reducing lignin accumulation and delaying hardness increase [52,53].

### 3.4. Modulation of q-PCR by HEER

**Figure 6:**
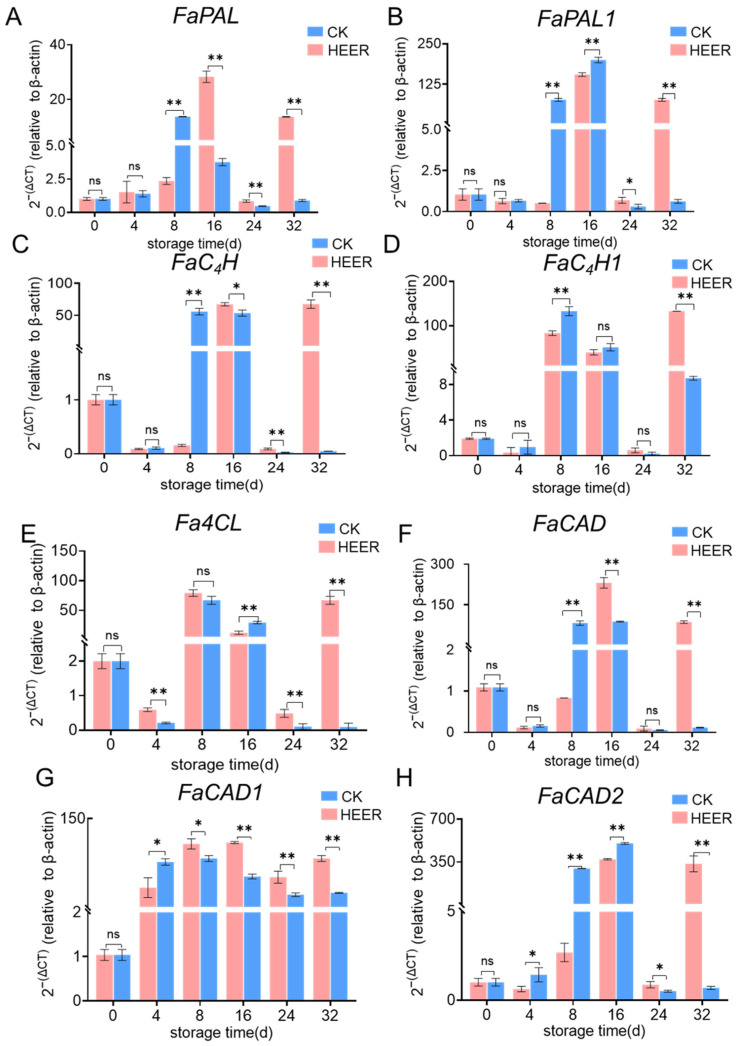
*FaPAL* (**A**), *FaPAL1* (**B**), *FaC_4_H* (**C**), *FaC_4_H1* (**D**), *FaC4L* (**E**), *FaCAD* (**F**), *FaCAD1* (**G**), and *FaCAD2* (**H**) were identified in faba bean samples treated with the HEER at 0 °C for a period of up to 32 days. (The main ingredient in HEER is potassium permanganate (KMnO_4_) in powder which is packaged in sachets (these sachets must have high permeability to gaseous ethylene while being impermeable to water). The purity of KMnO_4_ is 99% for analytical reagent grade. It showcases scavengers containing approximately 4–6% KMnO_4_ on an inert substrate.) (* *p* < 0.05, ** *p* < 0.01).

The expression patterns of 8 genes related to lignin synthesis in fresh faba beans were investigated, as shown in Figure 6. Numerous studies have demonstrated the significance of the phenylpropane metabolic pathway in enhancing postharvest fruit and vegetable hardness, which is crucial for storage quality. PAL, C_4_H, and 4CL are essential enzymes in this pathway, primarily responsible for producing most of the precursors of phenolics [42]. In the HEER group, *FaPAL* expression sharply increased after 8 days of storage, exhibiting a significant difference (4.8-fold) compared to the CK group. In the CK group, *FaPAL* expression peaked at day 16, then drastically decreased before increasing again at day 32 (Figure 6A), with differences also observed in HEER-treated beans (6.5-fold and 14.3-fold). The suppression of *FaPAL* expression by HEER during the late storage period (16–32 days) aligns with similar findings in postharvest kiwifruit treated with 1-MCP [50].

The expression peaks of HEER and CK of *FaPAL1* Figure 6B all appeared at day 16 of the storage period, with HEER inhibiting *FaPAL1* activity during days 24–32 of storage.

Ethylene induced a significant increase in the expression of *FaC_4_H* in fresh faba beans after 8 days of storage, which remained elevated at 16 days and then declined (Figure 6C). The peak expression of the CK group occurred at 16 days and remained high until the end of the storage period (day 32). Ethylene also decreased *FaC_4_H* expression during the middle and late stages of storage, and *FaC_4_H1* expression showed a similar trend to *FaC_4_H* (Figure 6D). During the pre-storage period, Fa4CL expression in HEER-treated faba beans increased, peaking at day 8 (Figure 6E). At day 16, *Fa4CL* expression was 1.39 times higher than CK. However, from days 24–32, *Fa4CL* expression in HEER was lower than CK. Xie et al. [12] found that 1-MCP treatment suppressed 4CL in postharvest kidney beans during late storage periods.

*FaCAD* expression in faba beans during storage initially increased and then gradually decreased (Figure 6F–H). For *FaCAD1*, the control level peak at day 16 was 1.1 times higher compared to the HEER group (Figure 6G). *FaCAD2* expression in HEER followed the same trend as *FaCAD1* (Figure 6H). Combined with enzyme activity results, these findings suggested that HEER may modulate the postharvest faba bean phenylpropane metabolic pathway through the decreased enzyme activity and down-regulation of *FaCAD1*, which was supported by the findings of Trabucco et al. [54]. The down-regulation of *FaCAD1* in Brachypodium distachyon was associated with a decrease in S monomers and a slight increase in G monomers, resulting in a lower S/G ratio, where CAD primarily consists of lignin composition [55,56].

As shown in Figure 4, the expression of *FaPAL* and *FaCAD* was highly similar to enzyme activity results, suggesting they play decisive roles among the eight genes studied. Based on the gene expression level, *FaPAL* was identified as the most important gene, followed by *FaCAD1*. Previous studies have reported strong expression of up to nine CAD-like genes during lignification in arabidopsis thaliana, particularly in vascular bundles [57,58,59]. It is well established that CAD and PAL play essential roles in lignin biosynthesis by facilitating lignin polymerization [41,52]. Overall transcriptomics results indicate that PAL and CAD genes may serve as key regulators of phenylpropane metabolism in postharvest faba bean treated with efficient ethylene removers, particularly highlighting the potential importance of *FaPAL* and *FaCAD1*.

## 4. Conclusions

The present study revealed the physiological indicators and associated gene expression in postharvest fresh faba bean treated with HEER. The application of HEER to fresh faba beans resulted in delayed vitamin C loss and maintained color quality. Notably, HEER treatment significantly reduced the increase in the hardness of the faba beans. This can be attributed to the maintenance of higher water content. Additionally, HEER treatment demonstrated an effect in suppressing the lignification of fresh faba beans, primarily by decreasing the activity of key enzymes involved in the phenylpropanoid metabolic pathway, including PAL, C4H, 4CL, and CAD. Furthermore, during storage, HEER treatment led to a reduction in the expression level of PAL and CAD genes. This resulted in a slower rate of CAD synthesizing lignin monomers and ultimately inhibited the increase in hardness. Notably, HEER lowers the expression level of PAL and CAD genes, especially *FaPAL* and *FaCAD1* genes, during storage. The former is the first rate-limiting enzyme in the phenylpropanoid metabolic pathway, and the latter is a key role in lignin synthesis. These findings indicated that the improved storage quality observed in postharvest fresh faba bean treated with HEER may be attributed to the combined effect of phenylalanine deaminase-related enzyme activities and gene expression.

## Figures and Tables

**Table 1:** Faba bean sequences for primers used in q-PCR analysis.

Gene Name	Forward Primer	Reverse Primer
*FaPAL*	5′-AGCAACACAACCAGGATGTCAA	5′-CAATTGCTTGGCAAAGTGCA
*FaPAL1*	5′-CTGGCACGACATCATAAGC	5′-GGAGGTGGTGGTGTTGTA
*FaC_4_H*	5′-CATTGAGCAGGGTTGTTGGC	5′-CCCACACATGAACCTCCACA
*FaC_4_H1*	5′-AGGCGAGATCAACGAAGACAAC	5′-GTTCACAAGCTCAGCAATGCC
*Fa4CL*	5′-AGGCAATGTACGTGGACAAGCT	5′-TCCGAGAGGACAGAGAAGTGGA
*FaCAD*	5′-CGAGTTGAACAGGGTCCGAA	5′-ATCAGCGTCCAATCCCACTC
*FaCAD1*	5′-AACATAACGAGTGACATTGAAT	5′-AACGGTAGCGAACATCAT
*FaCAD2*	5′-ATTGGCTGCAACTGACCCTT	5′-CAAGCATCTGGGTCGTGTCT
Actin gene	5′-GCGGACAGAATGAGCAAGGA	5′GAAGCCAAGATAGAGCCACCAAT

## Data Availability

The original contributions presented in the study are included in the article, further inquiries can be directed to the corresponding author.
